# Supplementary material for: Application of the digital annealer unit in optimizing chemical reaction conditions for enhanced production yields
Source: J Cheminform. 2025 Jul 14;17:105. doi: 10.1186/s13321-025-01043-y (PMC12261849; doi:10.1186/s13321-025-01043-y)
Supplement: Supplementary file 2 [file 13321_2025_1043_MOESM2_ESM.docx]

**Supplementary Information**

**Application of the Digital Annealer Unit in Optimizing Chemical Reaction Conditions for Enhanced Production Yields**

Shih-Cheng Li^+^, Pei-Hwa Wang^+^, Jheng-Wei Su, Wei-Yin Chiang, Tzu-Lan Yeh, Alex Zhavoronkov, Shih-Hsien Huang, Yen-Chu Lin, Chia-Ho Ou, and Chih-Yu Chen*
_____________________________________________________________________

^+^ These authors contributed equally.
^*^ Corresponding author

**Table of contents**

[**Supplementary Figure 1** 2](#_Toc185175854)

[**Supplementary Figure 2** 3](#_Toc185175855)

[**Supplementary Figure 3** 4](#_Toc185175856)

[**Supplementary Figure 4** 5](#_Toc185175857)

[**Supplementary Figure 5** 6](#_Toc185175858)

[**Supplementary Figure 6** 7](#_Toc185175859)

[**Supplementary Table 1** 8](#_Toc185175860)

[**Supplementary Table 2** 9](#_Toc185175861)

[**Supplementary Table 3** 10](#_Toc185175862)


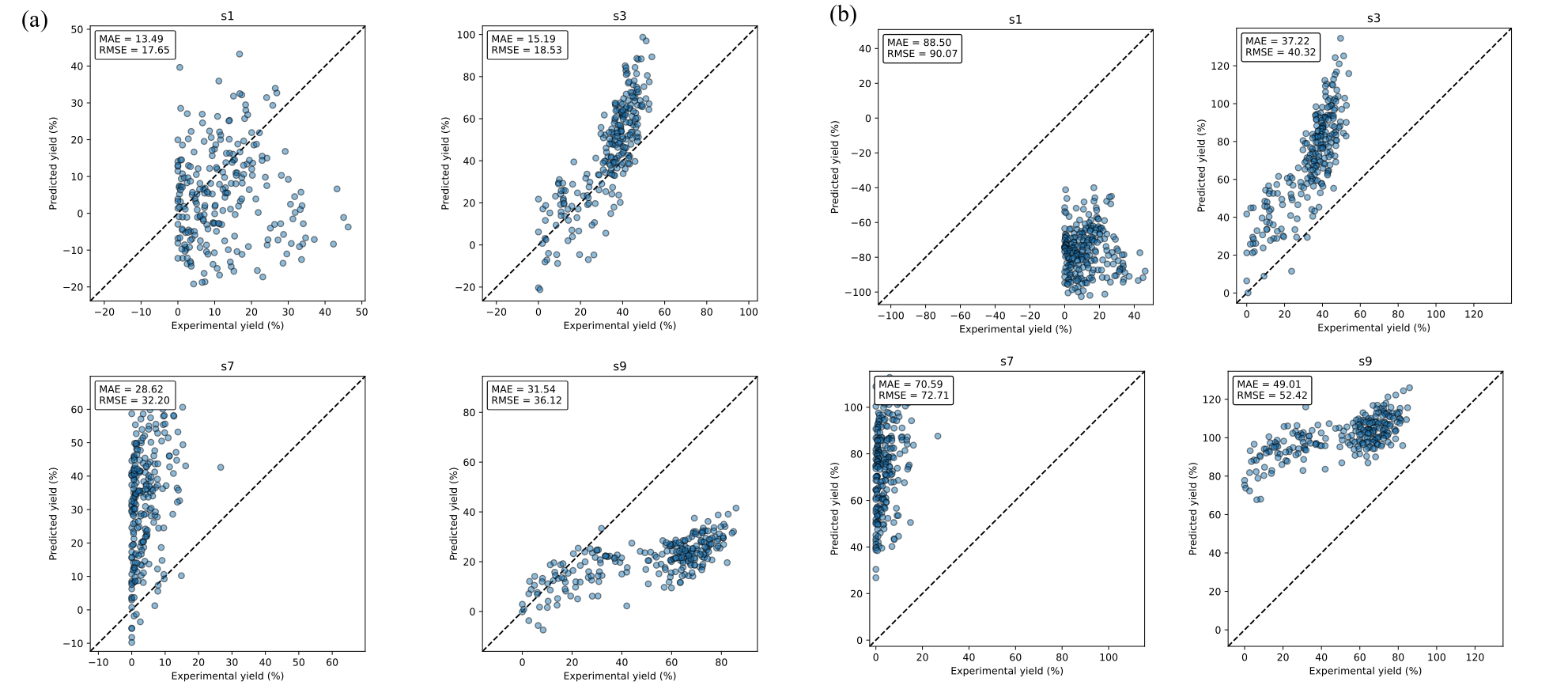


**Supplementary Figure 1** Results for the models trained on three subsets selected from s1, s3, s7, and s9, evaluated on the held-out test set of the C-N cross-coupling dataset via (a) ML-based and (b) DAU-based model.


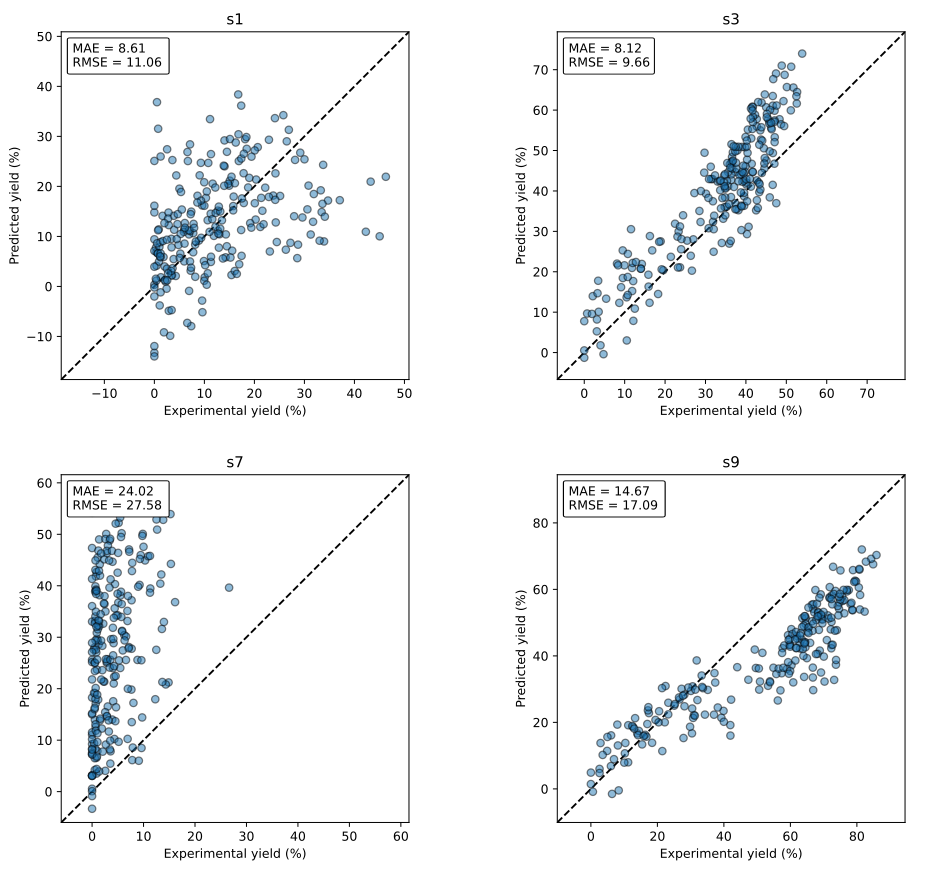


**Supplementary Figure 2** Results for the models trained on all subsets except for the held-out test set from the C-N cross-coupling dataset.


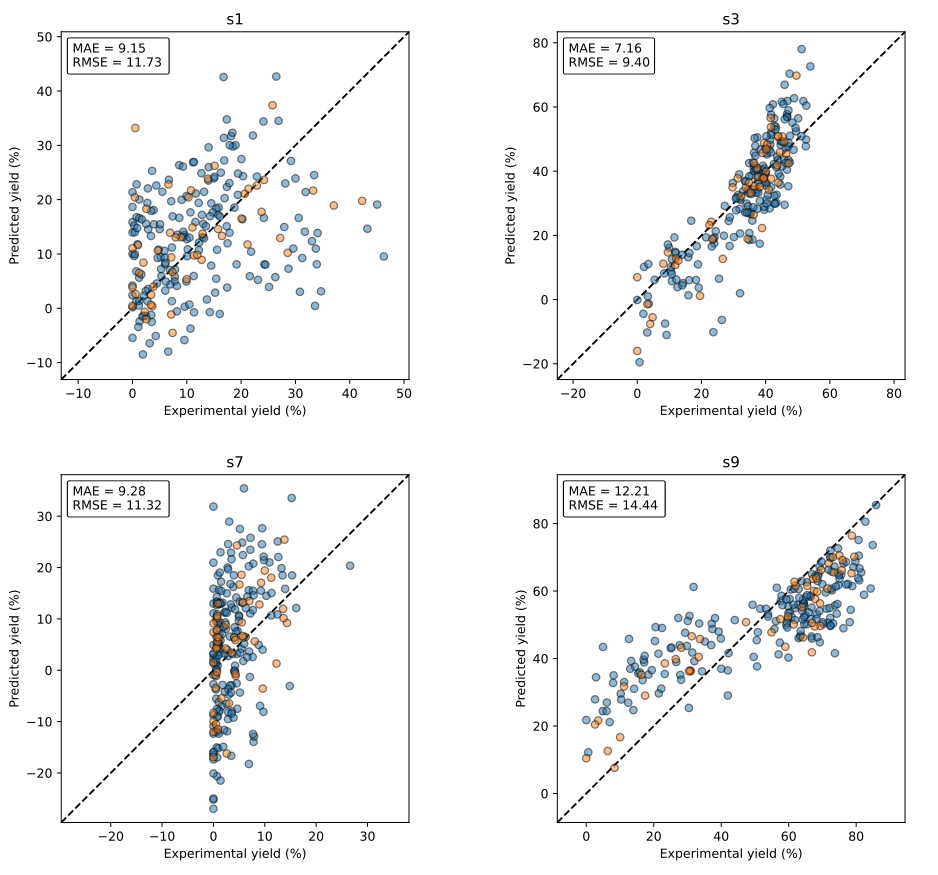


**Supplementary Figure 3** Results for the models trained on three subsets selected from s1, s3, s7, and s9, with an additional 50 data points from the held-out test set included in the training set, evaluated on the C-N cross-coupling dataset’s held-out test set. Orange points represent the test data included in the training set; all other points belong to the test set.


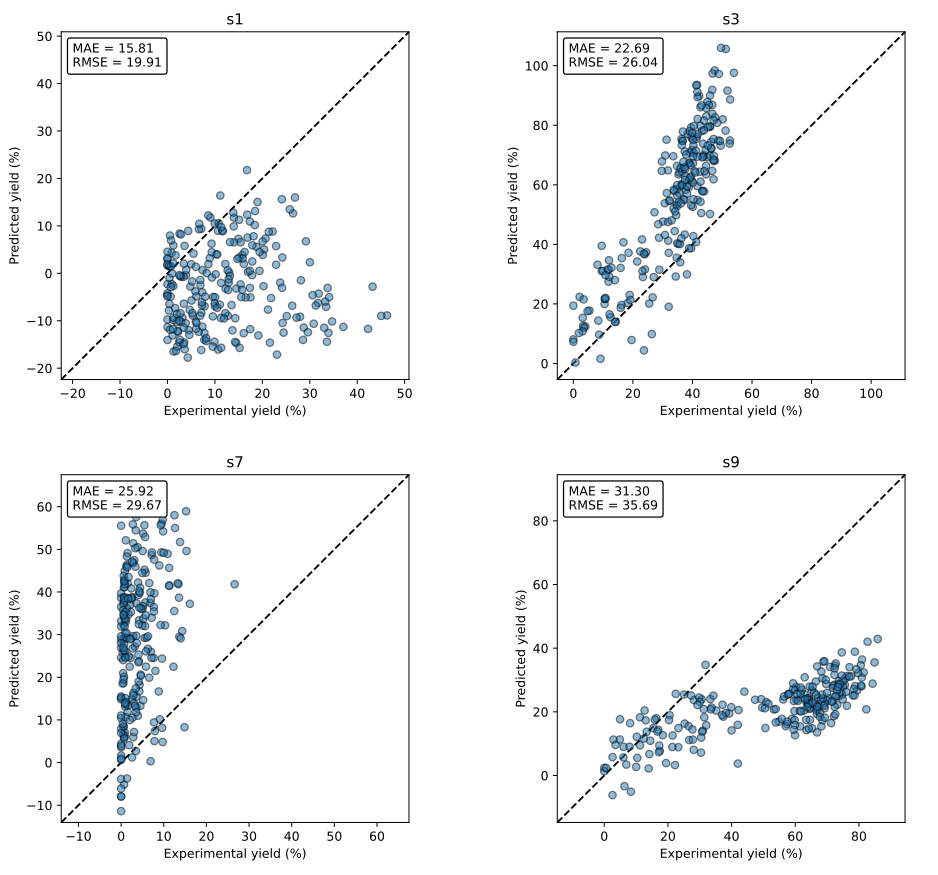


**Supplementary Figure 4** Results for the MLP models trained on three subsets selected from s1, s3, s7, and s9, evaluated on the held-out test set of the C-N cross-coupling dataset.


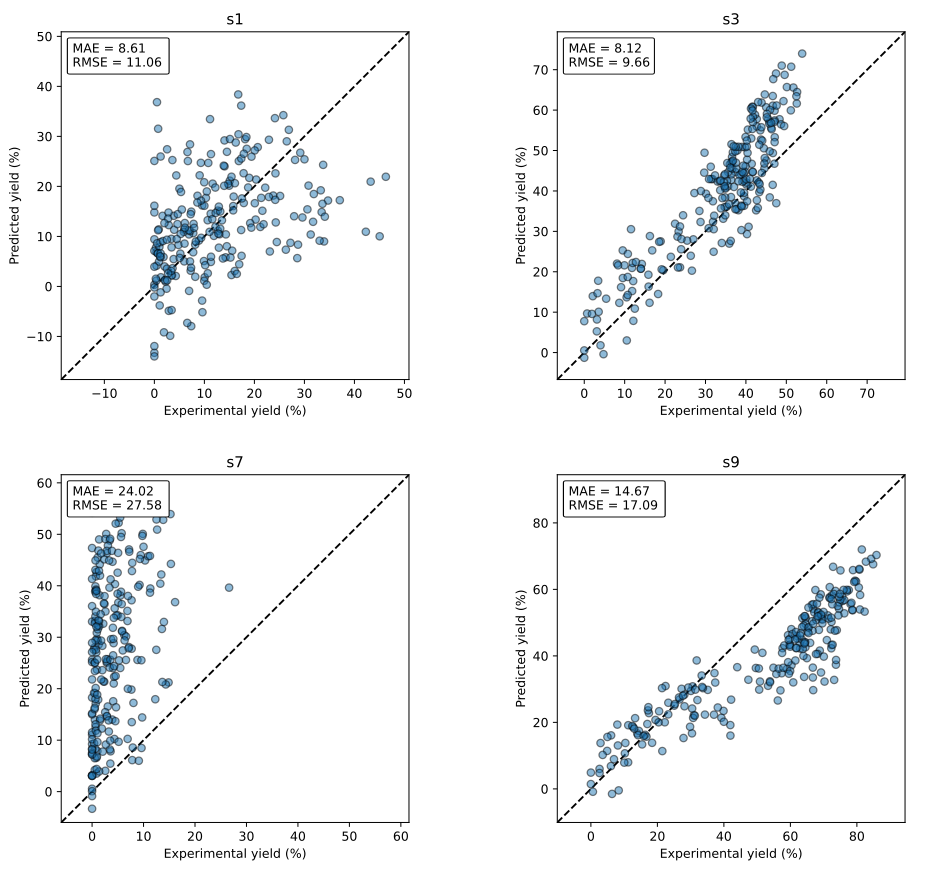


**Supplementary Figure 5** Results for the models trained on all subsets except for the held-out test set from the C-N cross-coupling dataset.


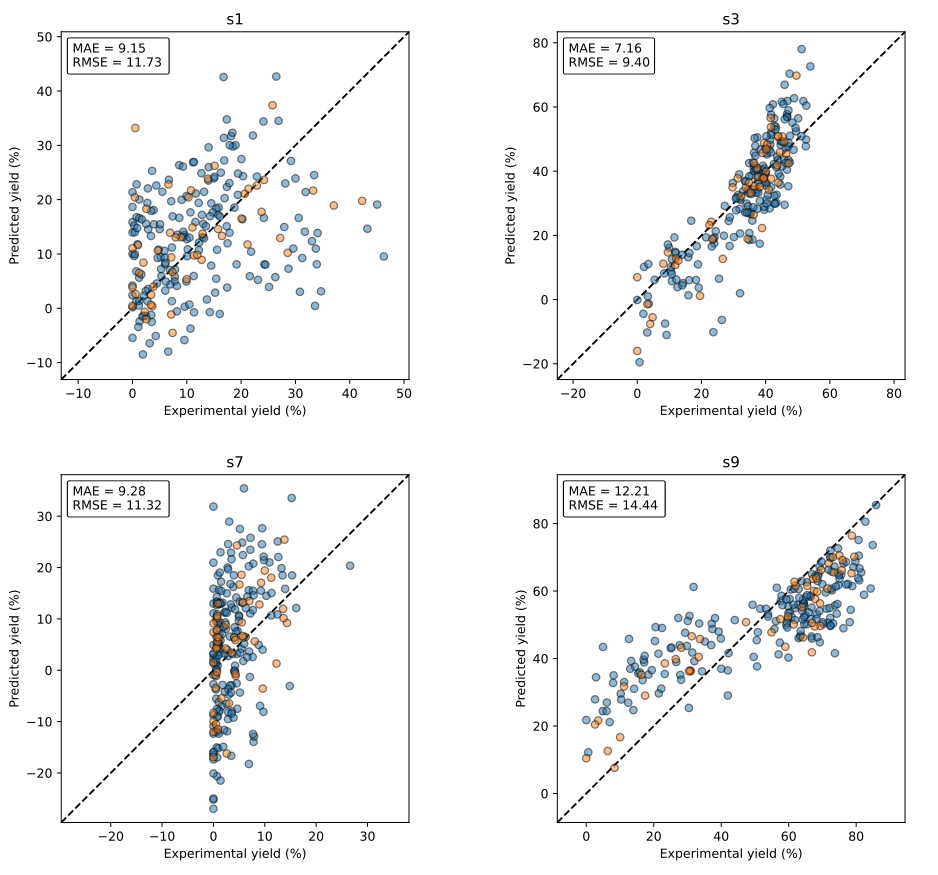


**Supplementary Figure 6** Results for the models trained on three subsets selected from s1, s3, s7, and s9, with an additional 50 data points from the held-out test set included in the training set, evaluated on the C-N cross-coupling dataset’s held-out test set. Orange points represent the test data included in the training set; all other points belong to the test set.

**Supplementary Table 1** Testing MAE for models trained on Buchwald-Hartwig C-N cross-coupling reactions, using various combinations of reaction encoding approaches and fingerprints, from the Reaxys database. The temperatures are either not specified, provided in evenly distributed bins, or presented in a binary format

|  | Evenly distributed bins | | |  | Binary encoding | | |  | No contribution | | |
| --- | --- | --- | --- | --- | --- | --- | --- | --- | --- | --- | --- |
|  | MACCS | Avalon | ECFP4 |  | MACCS | Avalon | ECFP4 |  | MACCS | Avalon | ECFP4 |
| reac_prod | 14.57 | 33.02 | 30.64 |  | 14.46 | 36.34 | 30.97 |  | 14.97 | 30.83 | 28.79 |
| reac_only | 13.59 | 17.70 | 16.25 |  | 13.55 | 17.50 | 17.27 |  | 13.71 | 17.63 | 16.24 |
| prod_only | 13.37 | 17.84 | 16.05 |  | 13.53 | 18.20 | 16.68 |  | 13.81 | 17.35 | 16.13 |
| reac_diff | 13.59 | 31.69 | 21.83 |  | 13.67 | 35.61 | 23.87 |  | 13.75 | 37.84 | 22.92 |
| prod_diff | 13.76 | 31.78 | 22.27 |  | 14.01 | 29.22 | 23.09 |  | 14.10 | 32.69 | 21.04 |
| diff_only | 14.15 | 15.20 | 14.37 |  | 11.59 | 13.60 | 11.64 |  | 14.59 | 15.83 | 14.73 |

**Supplementary Table 2** Testing MAE for models trained on Suzuki reactions, using various combinations of reaction encoding approaches and fingerprints, from the Reaxys database. The temperatures are either not specified, provided in evenly distributed bins, or presented in a binary format.

|  | Evenly distributed bins | | |  | Binary encoding | | |  | No contribution | | |
| --- | --- | --- | --- | --- | --- | --- | --- | --- | --- | --- | --- |
|  | MACCS | Avalon | ECFP4 |  | MACCS | Avalon | ECFP4 |  | MACCS | Avalon | ECFP4 |
| reac_prod | 14.81 | 40.87 | 46.17 |  | 15.38 | 44.06 | 46.38 |  | 14.64 | 43.54 | 45.13 |
| reac_only | 14.00 | 18.77 | 19.39 |  | 14.19 | 18.16 | 18.91 |  | 14.22 | 18.17 | 19.24 |
| prod_only | 13.97 | 19.36 | 17.92 |  | 13.92 | 19.64 | 18.58 |  | 14.00 | 18.92 | 17.93 |
| reac_diff | 14.51 | 41.97 | 31.75 |  | 14.40 | 46.72 | 33.23 |  | 14.67 | 40.62 | 30.80 |
| prod_diff | 14.32 | 40.95 | 27.85 |  | 14.30 | 40.37 | 28.63 |  | 14.25 | 42.27 | 28.11 |
| diff_only | 14.29 | 17.26 | 14.55 |  | 14.32 | 17.18 | 14.93 |  | 14.46 | 17.16 | 14.91 |

**Supplementary Table 3** Testing MAE for models trained on Negishi reactions, using various combinations of reaction encoding approaches and fingerprints with different bit sizes (128, 256, 512, 1024, and 2048), from the Reaxys database. The temperatures are not used as input in these models.

|  |  |  | Avalon |  |  |  |  |  | ECFP4 |  |  |
| --- | --- | --- | --- | --- | --- | --- | --- | --- | --- | --- | --- |
|  | 128 | 256 | 512 | 1024 | 2048 |  | 128 | 256 | 512 | 1024 | 2048 |
| reac_prod | 12.93 | 16.45 | 25.50 | 60.85 | 121.03 |  | 14.22 | 15.07 | 15.49 | 19.77 | 20.28 |
| reac_only | 11.65 | 12.43 | 14.74 | 21.66 | 31.65 |  | 11.90 | 11.97 | 12.65 | 12.45 | 12.43 |
| prod_only | 11.35 | 12.83 | 14.01 | 25.66 | 30.17 |  | 11.38 | 11.19 | 12.07 | 12.23 | 11.83 |
| reac_diff | 13.73 | 16.84 | 27.34 | 46.58 | 90.73 |  | 13.08 | 13.62 | 14.99 | 15.52 | 17.20 |
| prod_diff | 12.97 | 15.12 | 25.15 | 49.63 | 77.23 |  | 12.98 | 13.66 | 13.41 | 14.54 | 15.27 |
| diff_only | 11.94 | 12.76 | 13.76 | 14.36 | 19.67 |  | 11.94 | 11.16 | 10.98 | 11.30 | 11.58 |
